# Practice patterns in pediatric infectious encephalopathy in four centers in Africa

**DOI:** 10.3389/fped.2024.1304245

**Published:** 2024-02-23

**Authors:** Tigist Bacha, Alexandra Obremskey, Jessica Buxton, Ericka L. Fink, Amelie von Saint Andre-von Arnim, Madiha Raees

**Affiliations:** ^1^ Department of Pediatrics and Child Health, St. Paul Hospital Millennium Medical College, Addis Ababa, Ethiopia; ^2^ Department of Pediatrics, University of Washington, Seattle, WA, United States; ^3^ Department of Molecular and Cell Biology, University of California, Berkeley, CA, United States; ^4^ Department of Critical Care Medicine, UPMC Children’s Hospital of Pittsburgh, University of Pittsburgh School of Medicine, Pittsburgh, PA, United States; ^5^ Department of Pediatrics, Division of Pediatric Critical Care Medicine, University of Washington, Seattle Children’s, Seattle, WA, United States; ^6^ Department of Global Health, University of Washington, Seattle, WA, United States; ^7^ Division of Critical Care Medicine, Department of Anesthesiology and Critical Care, Perelman School of Medicine at the University of Pennsylvania, Philadelphia, PA, United States; ^8^ Division of Critical Care Medicine, Department of Anesthesiology and Critical Care, The Children’s Hospital of Philadelphia, Philadelphia, PA, United States

**Keywords:** pediatric, encephalopathy, meningitis, low middle income countries (LMICs), infectious diseases, child, cerebral abscess

## Abstract

**Introduction:**

Infectious encephalopathy (IE), including meningitis, infectious encephalitis, and cerebral abscess, remains prevalent and carries high mortality and morbidity in children, especially in low and middle income countries (LMIC). This study aims to describe the usual care and outcomes of pediatric IE in four LMIC hospitals in sub-Saharan Africa to support evidence-based care guideline development.

**Methods:**

This is a secondary analysis of the Prevalence of Acute Critical Neurological disease in children: A Global Epidemiological Assessment—Developing Countries study, a 4-week, prospective, observational study in children (1 week to 17 years) with IE presenting to referral hospitals in Ethiopia, Kenya, Rwanda, and Ghana. Data collection included diagnostic testing, interventions, and patient outcomes [e.g., mortality, Pediatric Cerebral and Overall Performance Category Scores (PCPC and POPC)].

**Results:**

Seventy-two children with IE were enrolled. Most patients were diagnosed with undifferentiated IE (78%, *n* = 56). Specific etiologies included cerebral malaria (10%, *n* = 7), viral encephalitis (4%, *n* = 3), tuberculosis (4%, *n* = 3), bacterial meningitis (3%, *n* = 2), and cerebral abscess (1%, *n* = 1). Fourteen patients (20%) had a head computed tomography performed. Thirty two (44%) children had a lumbar puncture but only 9 samples (28%) were sent for culture. Median time from diagnosis to antimicrobial therapy was 3 h (IQR 1–12 h). Half (51%, *n* = 33) of inpatients received intracranial pressure (ICP)-directed treatment but none underwent ICP monitoring. Mortality was 13% (*n* = 9). The percentage of children with a favorable cognitive score decreased from 95% (*n* = 62) prior to admission to 80% (*n* = 52) and 77% (*n* = 50) at discharge for PCPC and POPC respectively.

**Discussion:**

IE led to considerable morbidity and mortality in this cohort, and evaluation and management varied across the care continuum. Resource limitations and diagnostic constraints may have affected diagnosis-directed therapy and other aspects of management. Further studies are needed to describe the epidemiology and management of IE in LMICs to inform future treatment protocols, the role of technological and human capacity building to support both basic monitoring and interventions, as well as creative new solutions to emergency and critical care in these settings.

## Introduction

Infectious encephalopathy (IE), which includes meningitis, infectious encephalitis, and cerebral abscess, remains widely prevalent among children globally despite advancements in prevention. Meningitis and infectious encephalitis accounted for nearly 185,000 deaths and half a million DALYs in 2019 alone. The highest global burden of disease, morbidity, and mortality remains in low- and middle-income countries (LMIC) (1). Mortality due to infectious encephalopathy is twice as high in LMICs compared to high income countries (HICs) (2). With regard to morbidity, survivors of IE in childhood often experience incomplete recovery and many experience decreased quality of life (3–6).

Many factors likely contribute to the global disparities in outcomes. Bacterial meningitis is particularly morbid and has a high incidence in the “Meningitis Belt” in Sub-Saharan Africa stretching from Senegal to Ethiopia. LMICs have a higher prevalence of childhood HIV and malaria, and coinfection with IE confers worse outcomes in this patient population (5, 7–10). Poor healthcare infrastructure, sociopolitical instability, armed conflicts, as well as the challenge of accurately diagnosing neuro-infections, lead to delay in treatment and therefore poor outcomes in these settings (11, 12). International consensus statements and country-specific guidelines around meningitis and encephalitis (13–18) generally lack evidence specific to pediatric IE patients and typically do not account for resource constraints in LMICs. The World Health Organization does provide some high level guidance for meningitis care including antibiotic selection in endemic settings in Africa. However, the guidance does not provide specific management details (17). As a result, diagnosis and management of IE in these settings remain widely variable (19, 20).

This study aims to build on prior knowledge gained from the Prevalence of Acute Critical Neurological disease in children: A Global Epidemiological Assessment—Developing Countries (PANGEA-DC) study and further evaluates usual care, diagnosis, management, and outcomes of pediatric IE in four public referral hospitals in Ethiopia, Kenya, Rwanda, and Ghana (2).

## Materials and methods

This is a secondary analysis of PANGEA-DC, which was a 4-week, prospective, observational study in children with IE and traumatic brain injury conducted in 2017. Study sites included four public referral hospitals: Tikur Anbessa Specialized Hospital, in Addis Ababa, Ethiopia; Kenyatta National Hospital, in Nairobi, Kenya; University Teaching Hospital of Kigali, Rwanda; and the Wenchi Methodist Hospital, in Kumasi, Ghana. Study sites were selected as a convenience sample based on existing relationships with the PANGEA research group.

The observational study and data coordinating center (Pittsburgh, PA) were approved by the Institutional Review Board at the University of Pittsburgh, and local regulatory approval was obtained at each study site. Each site was a public referral center with a locally available emergency medical system. A limited number of narrow clinical protocols existed for pediatric patients with IE at the study sites (18). All centers were able to provide noninvasive and invasive ventilation, perform routine laboratory studies, and had head computed tomography (CT) scanners on site.

Children aged 1 week to 17 years presenting to the emergency department with either suspected or confirmed IE were included in this analysis. Participants meeting these criteria were identified by site investigators who provided clinical care or consultation at their respective centers. Data collection occurred using completion of a case report form (CRF) utilizing manual paper chart review and included patient and center demographics, diagnostic testing, medical interventions, and patient outcomes. Specifically, laboratory and brain imaging studies performed in the first 24 h of hospitalization, including cultures, organ supports, and monitoring, treatment for suspected intracranial hypertension (sedatives, analgesics, and hyperosmolar therapy) were specifically queried; not all therapies were available for each patient at each center on a given day. The data was de-identified and transcribed from the CRF to a central database at the University of Pittsburgh Data Coordination Center.

Patient outcomes included hospital mortality and change in Pediatric Cerebral Performance Category (PCPC) and Pediatrics Overall Performance Category (POPC) scores from prior to admission. The PCPC and POPC scores are standardized assessments commonly used to quantify new disability following illness. All scores were calculated by the center investigators; pre-illness scores were assigned based on caregiver interview, and discharge scores were assigned using the medical chart. A favorable score was considered a score of 1 or 2, consistent with no or very mild disability. A score of 6 is consistent with death (21). For the purposes of comparison testing, an unfavorable outcome was defined as a new discharge PCPC or POPC score >2, mortality, and/or a new morbidity (new feeding tube, new tracheostomy tube, hydrocephalus, dysautonomia, or new nosocomial infection).

The primary objective was to report monitoring, testing, therapeutics, and outcomes for children with IE presenting to one of the four hospitals in Sub-Saharan Africa as patients traveled through the care continuum.

Descriptive statistics were reported as median with interquartile range (IQR) as the data set was non-parametric. Less than 10% of the data was missing and therefore not imputed; thus, resulting in variable sample sizes for certain parameters. Data were compared using Chi-square testing, Mann–Whitney testing, Wilcoxon rank sum, and Spearman's correlation as appropriate. All *p-*values less than 0.05 were considered statistically significant. Stata software (College Station, TX) was used for the analysis.

## Results

Seventy-two children with IE were enrolled on hospital presentation and followed until their discharge. The median age was 6 months and 49% (*n* = 35) of patients were female (Table 1). Of these patients, 42 (58%) were enrolled at the Kenyan site, 15 (21%) in Ethiopia, 8 (11%) in Rwanda, and 7 (10%) in Ghana. The majority of patients (72%, *n* = 52) had no reported past medical history.

**Table 1:** Patient demographics.

Variable	Overall, *n* = 72, *n* (%)
Age (months)	6 (1–36)
Female	35 (49)
Type of infection	
Infectious encephalopathy, unspecified	56 (78)
Encephalitis	3 (4)
Abscess	1 (1)
Cerebral malaria	7 (10)
Mycobacterium tuberculosis meningitis	3 (4)
Bacterial meningitis	2 (3)
Previously healthy	52 (72)
Past medical history	20 (28)
Prematurity	3 (4)
Congenital heart disease (unrepaired)	2 (3)
Seizure disorder	2 (3)
Cerebral palsy	2 (3)
HIV	2 (3)
Pulmonary tuberculosis	3 (4)
Study site	
Kenya	42 (58)
Ethiopia	15 (21)
Rwanda	8 (11)
Ghana	7 (10)

Fifty-six (78%) patients had no etiologic infectious organism or process identified as the cause of IE. Of those that did, the most common cause was cerebral malaria (*n* = 7), followed by unspecified viral encephalitis (4%, *n* = 3) and tuberculous meningitis (4%, *n* = 3).

In the emergency department (ED), 36% (*n* = 26) of patients had a complete set of vital signs including temperature, heart rate, respiratory rate, blood pressure, and oxygen saturation recorded. The most commonly obtained vital sign was temperature (70%), and the least common was oxygen saturation (36%). For those who had any vital sign recorded (*n* = 51), 45% (23/51) were febrile, 4% (2/51) were hypothermic, 46% (12/26) were hypoxic, 72% (21/29) were tachycardic for age, and 2% (1/44) were hypotensive for age by World Health Organization (WHO) criteria. A Glasgow Coma Scale (GCS) score was completed on 22 patients in the ED with a median score of 14.5 (IQR 11–15). A Blantyre coma scale (BCS) score for preverbal children was completed on 7 patients with a median score of 5 (IQR 4–5). A lumbar puncture (LP) was performed in 44% of patients (*n* = 32), and 20% (*n* = 14) had a head computed tomography (CT) performed. Six of these patients (6/14, 43%) had evidence of abscess or infection on CT; the remainder were reported as normal. Laboratory work was performed on the majority of patients (90%, *n* = 65), with a full blood count as the most common investigation. Sixty-nine percent (*n* = 50) had a serum sodium value checked, with more than half (58%, *n* = 29/50) of these results revealing hyponatremia (sodium <135 mEq/L). Blood cultures were sent in 8% (*n* = 6) of patients and cerebrospinal fluid (CSF) cultures in 13% (*n* = 9) of patients.

No vasopressors or inotropes were initiated in the ED. Thirteen patients (18%) required oxygen delivered via simple face mask or nasal cannula. Six patients (13%) required bag-mask ventilation temporarily at some point during their ED evaluation. One patient was placed on non-invasive positive pressure ventilation in the ED. In total, 65 (90%) patients were admitted to the hospital; the majority (86%, *n* = 62) to the general pediatric ward, while 4% (*n* = 3) were admitted to the pediatric intensive care unit (PICU).

Time to antimicrobial therapy from diagnosis was documented for 92% of patients (*n* = 62) admitted to the hospital. Median time to receive an antimicrobial was 3 h (IQR 1–12 h, Range 0–80 h). A third of patients (33%, *n* = 21) received antimicrobial therapy within 1 h of diagnosis.

Continuous pulse oximetry and cardiovascular monitoring were not used on any ward or PICU patients. One PICU patient required vasoactive medications, and thus had a central venous catheter placed; no arterial catheters were placed. Two PICU patients required endotracheal intubation and invasive mechanical ventilation; median duration of mechanical ventilation was 2.5 days (IQR 1–4). A standard electroencephalogram (EEG) was performed for abnormal movements in one ward patient and was negative for seizures; another was performed in one PICU patient and was consistent with focal seizures. No patient had continuous EEG or brain magnetic resonance imaging performed.

**Figure 1:**
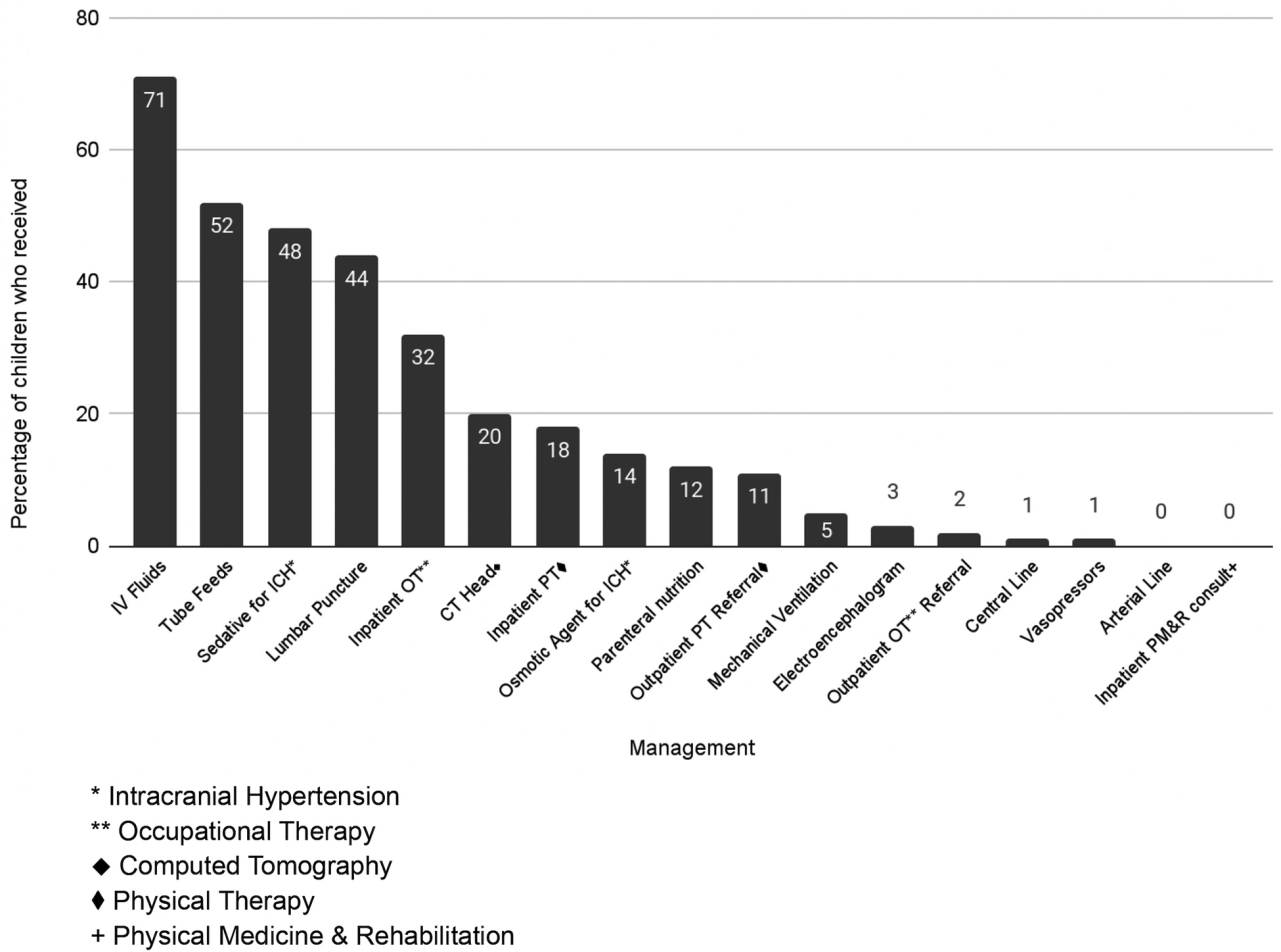
Frequency of management and interventions provided.

Around half (51%, *n* = 33) of inpatients received intracranial pressure (ICP)-directed treatment, however ICP monitoring was not available. The most common therapy was in the form of sedation/analgesia (49%, *n* = 32); seven patients received hyperosmolar therapy for presumed increased ICP (mannitol *n* = 2, hypertonic saline *n* = 5) (Figure 1). Nine patients (14%) received multiple medical therapies to decrease ICP. No patients received a decompressive craniectomy.

Median length of stay was 7 days (IQR 2–30) with median ICU length of stay 4.5 days (IQR 1–5 days). The majority of inpatients were discharged to home (83%, *n* = 54); nine children died. Two patients from Kenya were discharged elsewhere; one to an inpatient rehabilitation unit and another to a skilled nursing facility. Fifteen percent (*n* = 20) of children left the hospital with a new feeding tube, and 17% (*n* = 11) had new hypertonia/spasticity. Six percent of children (*n* = 4) had new hydrocephalus, two of whom required surgical ventriculoperitoneal shunt placement (Table 2).

**Table 2:** Medical management and outcomes.

Variable	*n* (%) or median (IQR)
Prehospital care	
None	37 (51)
BLS	33 (46)
Advanced life support (physician present)	2 (3)
Transport to tertiary center	
Transport distance (km)	15 (9–18)
Transport duration (minutes)	60 (30–60)
Presenting characteristics in ED	
Loss of consciousness	29 (53)
Seizures	44 (62)
Hypotension	1 (2)
Fever	23 (45)
Hypothermia	2 (4)
Hypoxia	12 (46)
Tachycardia	21 (72)
Blantyre coma score, *n* = 7	5 (4–5)
Glasgow coma score, *n* = 22	14.5 (11–15)
ED respiratory support	
Room air	51 (71)
Oxygen	13 (18)
Required bag-valve mask	6 (8)
≤1 h to antimicrobial therapy	21 (33)
Hospital complications	
Dysautonomia	3 (5)
Hydrocephalus	4 (6)
Hypertonia/spasticity	11 (17)
Feeding tube	20 (15)
Tracheostomy	0 (0)
Nosocomial infection	4 (6)
Disposition at discharge	
Home	61 (85)
Inpatient rehabilitation center	2 (1.5)
Mortality	9 (13)

**Figure 2:**
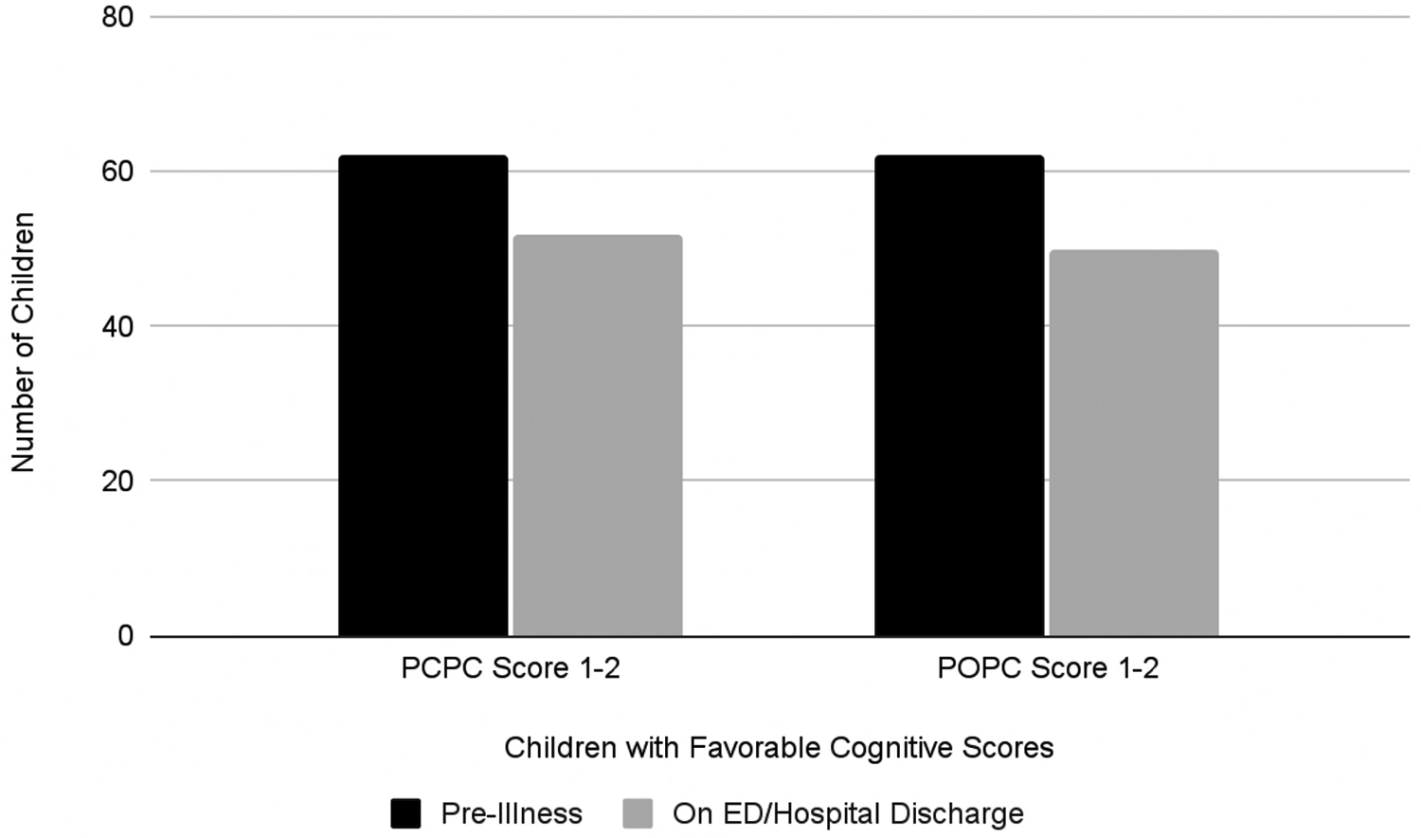
Progression of PCPC and POPC scores.

Overall mortality was 13% (*n* = 9). Most children (95%, 62/65) had a favorable pre-illness PCPC score of 1 or 2. At hospital discharge, this proportion decreased to 80% (*n* = 52) with 44 children who scored 1, 8 scored 2, 2 scored 3, 1 scored 4, 1 scored 5, 9 scored 6 (Figure 2). The distribution and change in POPC score was similar; again, the majority (95%, *n* = 62) had a favorable pre-illness POPC score. At hospital discharge, the percentage of children with a favorable POPC score decreased to 77% (43 children scored 1, 7 scored 2; 4 children scored 3, 1 scored 4, 1 scored 5, and 9 scored 6 (Figure 2).

Physical therapy (PT) was available to 86% (*n* = 56) of inpatients and was utilized by 18 patients. Similarly, occupational therapy (OT) was available to 49% (*n* = 32) inpatients and utilized by 13 patients. Of the four survivors who had a worse PCPC score on discharge compared to pre-illness, two received PT; none received OT.

There was no association between hyponatremia (*p* = 0.694), anemia (*p* = 0.44), or acidosis (*p* = 0.08) and an unfavorable outcome. Additionally, there was no difference in outcome when comparing patients who received any ICP-directed therapy against those who did not (*p* = 0.113) and when comparing those who received lumbar punctures against those who did not (*p* = 0.061). There was a weak positive correlation (*r* = 0.222) between discharge PCPC score and length of stay.

## Discussion

The PANGEA-DC program assessed access to care, treatments, and outcomes in neurocritical conditions with a focus on four countries in sub-Saharan Africa. This secondary analysis of PANGEA-DC data provided a deeper insight into management variability and system limitations in care of children with IE in these settings.

This analysis has demonstrated (1) inconsistent utilization of available diagnostic tools, including vital sign measurements, GCS, lumbar puncture for CSF sampling, laboratory workup including blood and CSF cultures, and brain imaging, (2) frequent treatment of intracranial hypertension (ICH) without the guidance of ICP monitoring, and (3) a substantial increase in neurological morbidity at discharge in the face of minimal use of physical and occupational therapy.

The majority of children in this cohort had unspecified encephalitis and no identified organism. In fact, most patients had no available culture data which limits diagnosis-directed therapy. However, empiric antimicrobial therapy was started in a timely fashion in most cases. Prompt administration of antibiotics when IE is suspected is well accepted as standard of care. However, there is a lack of consensus around a benchmark time frame for initiating antimicrobial therapy reflected in the variability between official recommendations and vague guidance in the WHO meningitis guidelines (14, 17, 22, 23). Several high quality studies have demonstrated negative impacts on mortality and outcomes when antibiotics are delayed beyond the first several hours of presentation (24–26). Furthermore, sepsis can be a comorbid process in IE patients and there are well established Surviving Sepsis guidelines with a goal of antimicrobial administration within 1 h (27). Future international guidelines for IE should likewise include evidence-based timeframes for delivery of antimicrobial therapy.

Interestingly, 90% of patients had some lab work completed and 44% of patients underwent lumbar puncture, indicating that there is capacity to obtain routine studies. This demonstrates the possibility of standardizing care to allow for a blood and CSF evaluation in cases of suspected IE which would include an infectious workup with cultures, while recognizing that good quality microbiology faces significant infrastructural, technical, and human resource challenges in many hospitals in LMICs. Consensus guidelines for management of IE patients generally do not recognize such limitations and therefore have less utility in these settings (13–17).

While brain imaging was less common (20%) for diagnosis, it can provide valuable information as it did in our patients when abscesses/other localized and loculated infections could be detected. In neonates and infants (with an open anterior fontanelle), cranial ultrasound can be a useful and inexpensive diagnostic method for suspected bacterial meningitis. Ultrasound machines are available at many centers in LMIC, and ultrasonography is a fast and safe procedure (28, 29). Sonographic abnormalities are observed in approximately 65% of pediatric patients with uncomplicated bacterial meningitis and up to 100% in children with bacterial meningitis and severe neurological symptoms (30). Ultrasound and doppler imaging may help provide a quick preliminary diagnosis for initiation of treatment, which can have a significant prognostic impact (31). Current guidelines recommend magnetic resonance imaging (MRI) and CT which are not always easily available in LMICs. Thus, we would advocate for ultrasound to be included in the list of brain imaging modalities as adjunct diagnostic support of IE if available.

We recognize however that clinical assessment and basic bedside monitoring can sometimes be the only available monitoring techniques in some resource-limited settings. The GCS represents a viable alternative to advanced imaging or invasive monitoring as it is easy to learn, reliable with training, and recognised internationally. In children under age 5 years, the modified Glasgow Coma Scale or the BCS can be used (32).

Another notable finding of our study was the use of ICP-directed therapy in the absence of ICP monitoring. Practitioners reported using sedatives, analgesics, and hyperosmolar therapy (mannitol or hypertonic saline). It is not uncommon for practitioners in LMICs to utilize clinical judgment or non-invasive strategies to treat suspected intracranial hypertension, given lack of access to advanced technologies like continuous ICP monitoring. Investigators in South America report similar outcomes in using serial imaging as compared to ICP monitor use in severe traumatic brain injury (33). Ultrasound measuring optic nerve sheath diameter was used successfully in India for the identification of raised intracranial pressure in patients with tuberculous meningitis (28). Distinct changes in transcranial doppler (TCD) measurements were identified in African children with cerebral malaria that permitted phenotypic grouping associated with neurologic outcomes (34). However, robust evidence is required to demonstrate that techniques such as TCD and optic nerve sheath diameter identified with ultrasound detect raised intracranial pressure earlier, lead to appropriate interventions, and then improve outcomes.

Notably, both mortality and morbidity of our study patients was substantial with almost one third leaving the hospital with a new medical device (feeding tube or ventriculoperitoneal shunt). It has been suggested in HIC literature that earlier intervention with neurologically focused physical, occupational, and speech therapy can improve recovery and outcomes for patients following brain injury (35, 36). Access to and utilization of rehabilitative therapies was not consistent for our patient population which possibly contributed to the proportion of patients with an increase in their PCPC and POPC scores. Our study sites reflect an ongoing access issue to rehabilitative services in LMIC.

### Limitations

Data collected for this study came from referral centers in four LMICs over a 1 month time frame. As a result, this data may not be generalizable to less-resourced community hospitals. In addition, the data does not capture patients who either had mild cases and did not present to care or those who died prior to transfer to tertiary care. Infectious diseases, especially vector-borne diseases are more common in Sub-Saharan Africa, and are often seasonal. The short data collection period may have missed common pathologies at different times of year. Unfortunately, a longer study duration was limited by the available funding. Limited diagnostic availability led to difficulty confirming the diagnosis of IE and identifying specific pathogens. Furthermore, some aspects of management reported in this study would be specific to certain pathologies and contraindicated in others, such as a lumbar puncture, making it difficult to compare practice patterns. The study did not allow for data collection on daily changes in physical exam or vital signs which limited full understanding of patient progression. Data on specific antimicrobial therapies was not available, which made it challenging to evaluate appropriateness of empiric or targeted therapy and limited generalizability on patient outcomes. Data on use of adjunctive therapy such as steroids was also not available. This study also had a small “n” with limited documentation and no long term follow up due to its point prevalence design. Longer term outcomes for patients such as changes to PCPC and POPC scores, evolving morbidity, readmission rates or post-discharge mortality are not represented here. The study did not include infants in the first week of life which may have excluded a large proportion of perinatally acquired IE.

## Conclusion

Infectious encephalopathy is a common childhood disease in LMIC that can have devastating consequences. HICs have developed systems and clinical protocols around the management and treatment of IE, but in LMICs, evidence for such protocols and systems is more limited. The variability in care and the lack of resource use despite availability demonstrated in this study further underscore the need for such protocols. Resource limitations are a reality in LMIC care settings which was reflected in our data. Further studies are needed to describe the epidemiology and management of IE in LMICs to inform future treatment protocols, the role of technological and human capacity building to support both basic monitoring and interventions, as well as creative new solutions to emergency and critical care in these settings.

## Data Availability

The raw data supporting the conclusions of this article will be made available by the authors, without undue reservation.
